# Behavior and Distribution of Heavy Metals Including Rare Earth Elements, Thorium, and Uranium in Sludge from Industry Water Treatment Plant and Recovery Method of Metals by Biosurfactants Application

**DOI:** 10.1155/2012/173819

**Published:** 2012-05-27

**Authors:** Lidi Gao, Naoki Kano, Yuichi Sato, Chong Li, Shuang Zhang, Hiroshi Imaizumi

**Affiliations:** ^1^Graduate School of Science and Technology, Niigata University, Niigata 950-2181, Japan; ^2^Department of Chemistry and Chemical Engineering, Faculty of Engineering, Niigata University, Niigata 950-2181, Japan

## Abstract

In order to investigate the behavior, distribution, and characteristics of heavy metals including rare earth elements (REEs), thorium (Th), and uranium (U) in sludge, the total and fractional concentrations of these elements in sludge collected from an industry water treatment plant were determined and compared with those in natural soil. In addition, the removal/recovery process of heavy metals (Pb, Cr, and Ni) from the polluted sludge was studied with biosurfactant (saponin and sophorolipid) elution by batch and column experiments to evaluate the efficiency of biosurfactant for the removal of heavy metals. Consequently, the following matters have been largely clarified. (1) Heavy metallic elements in sludge have generally larger concentrations and exist as more unstable fraction than those in natural soil. (2) Nonionic saponin including carboxyl group is more efficient than sophorolipid for the removal of heavy metals in polluted sludge. Saponin has selectivity for the mobilization of heavy metals and mainly reacts with heavy metals in F3 (the fraction bound to carbonates) and F5 (the fraction bound to Fe-Mn oxides). (3) The recovery efficiency of heavy metals (Pb, Ni, and Cr) reached about 90–100% using a precipitation method with alkaline solution.

## 1. Introduction

With the rapid development of industry, a large quality of industrial sludge is settled down in wastewater treatment plants (WWTPs) every year. The sludge must be treated and disposed in a safe and effective manner because it may be contaminated with toxic organic and inorganic compounds. Much of this sludge is treated using a variety of digestion techniques to reduce the amount of organic matter and the number of disease-causing microorganisms, then the nutrient-rich sludge is provided to use as agricultural soil for landscaping and garden planting or as natural fertilizer [1–3]. These techniques have reduced the amount of landfill and changed waste into resource [4, 5]. However, the digested sludge cannot be directly used for practical use because it may contain hazardous inorganic substances such as heavy metals and radioactive elements. For this reason, it is of significant importance to investigate the removal of these metals by eco-friendly methods and to study the behavior and distribution of heavy metals in sludge from an environmental protection and human health perspective. On the other hand, the demand for trace metals such as rare earth elements (REEs) in modern society has increased markedly in recent years. The shortage of trace metals including REEs and uranium (U) has been of concern, and the investigation of new sources of these trace metals is important from a resources recovery point of view.

In recent years, the concentrations and distribution of heavy metals in sludge has been extensively studied [6–10]. Furthermore, the investigations of methods for removing of heavy metals from sludge have been widely carried out [11–14].

Total concentrations and fractions of heavy metals in sewage sludge from municipal and industrial wastewater treatment plants have been studied [6]. The results showed that the total concentrations of heavy metals in sludge varied greatly and that there was no significant difference in total metal concentration between municipal and industrial wastewater treatment plants. Chen et al. [7] reported the bioavailability and eco-toxicity of heavy metals in municipal sludge by taking into consideration both the speciation of metals and the local environmental characteristics. From this work, it was found that only the sludge from Xia Wan sewage treatment plant showed elevated concentrations of heavy metals and that the sludge from other plants showed low total concentrations of heavy metals except for a slightly higher concentration of Cd. The results of the sequential extraction procedure showed that Cu and Zn were principally distributed in the oxidize fraction and that Pb was mainly in the residual fraction. Furthermore, the different types of sludge and the distribution of the heavy metals in sludge have been studied [8]. It was confirmed that the total concentrations of heavy metals did not exceed the limits set out by the European legislation and that the stabilization method undergone by the sludge strongly influenced the distribution and the associated phases of heavy metals. The extractable forms of heavy metals in sludge from wastewater treatment plants have been determined to obtain suitable information about their bioavailability or toxicity [9]. In regard to current international legislation on the use of sludge for agricultural purposes, the concentrations of any metal did not exceed permitted levels. For most of the subject metallic elements, the increase of the concentrations was clearly found in two less-available fractions (oxidizable fraction and residual fraction) with the sludge treatment. In contrast, Ščančar et al. [10] determined the total and fractional concentrations of Cd, Cr, Cu, Fe, Ni, and Zn in sewage sludge samples from an urban wastewater treatment plant and showed that the sludge could not be used in agriculture due to the high total Ni concentration and its high mobility.

Currently, the removal of ultrasond-assisted metals from sludge is applied widely. For example, Deng et al. [11] and Li et al. [12] investigated the removal or recovery of heavy metals from sludge using ultrasound-assisted acid. The results showed that ultrasonic treatment is a necessary and effective method for assisting the improvement of heavy metal removal. However, ultrasonic treatment has an effect on the physical and chemical properties of sludge to some extent and is energy-consuming. In another study, Babel and Mundo Dacera [13] reviewed various methods for the removal of heavy metal from sewage sludge, including chemical extraction, bioleaching, electroreclamation, and supercritical fluid extraction (SFE). They compared the advantages and limitations of each and gave a detailed analysis of their findings. A combination of two methods (i.e., bioleaching and electrokinetic remediation technology) for removing heavy metals from sludge has been also reported [14]. The combined technology can not only remove the heavy metals in the sludge but also make them be recycled, although it is energy-consuming to some extent.

As mentioned above, most research has been mainly focused on toxic heavy metallic elements such as Cd, Pb, Cu, and Cr as subject elements and on the differences and characteristics of these elements according to different types of sludge or different treatment processes. However, few reports have been published about the behavior and distribution of REEs, Th, and U. Moreover, there have been very few comparisons between concentrations of heavy metals in sludge and those in natural soil carried out. It is important to compare the concentration and distribution of metals in sludge with those in natural soil when considering the utilization of sludge as agricultural soil in the future. The purposes of this paper are (1) to investigate the behavior, distribution, and characteristics of heavy metals including REEs, Th, and U in sludge compared with those in natural soil and (2) to study the removal/recovery process of heavy metals from polluted sludge with biosurfactant elution by batch and column experiments.

## 2. Experimental

### 2.1. Apparatus and Reagents

An inductively coupled plasma mass spectrum (ICP-MS) instrument (Thermo scientific X-Series) was used to determine the concentrations of REEs, Th, and U, and an inductively coupled plasma atomic emission spectrophotometer (ICP-AES) instrument (SPS1500, Seiko Instruments Inc.) was employed to determine the concentrations of heavy metals (Zn, Cd, Pb, Cr, Ni, and Cu). The operating conditions of the ICP-MS are the same as shown in our previous paper [15] and those of ICP-AES are based on our other previous paper [16].

Heavy metal standard solutions, including REEs, Th, and U, were purchased from SPEX CertiPrep, Inc. (USA). Each working standard solution was prepared by diluting the original standard solution.

**Figure 1:**
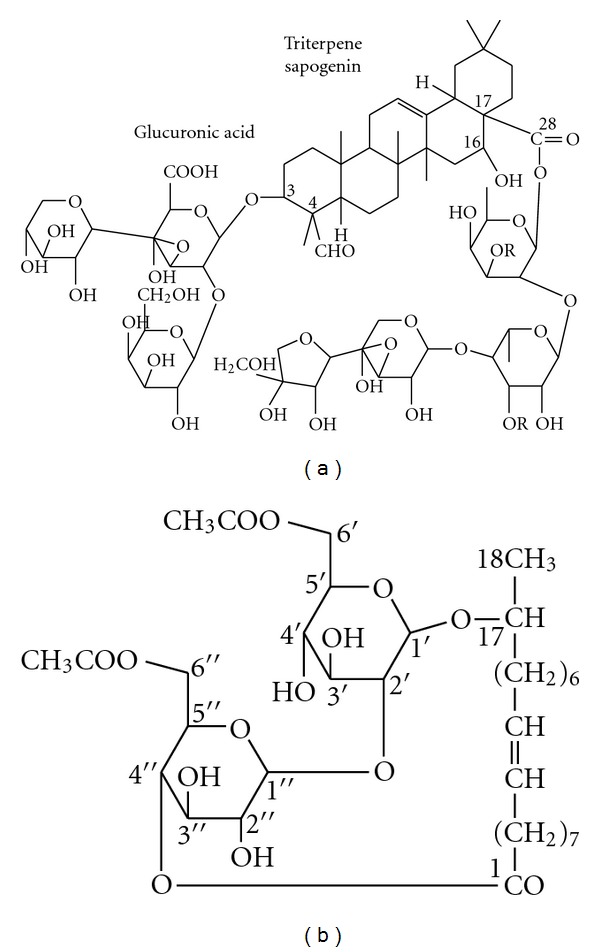
One structure of saponin (15) and sophorolipid (16).

In this work two kinds of biosurfactants were used, saponin and sophorolipid. Saponin was purchased commercially from Sigma-Aldrich, Inc. (Germany). It is a nonionic biosurfactant but includes the carboxyl group (–COOH) as shown in Figure 1(a) based on the analysis of *quillaja bark *by Guo and Kenne [17]. Sophorolipid was supplied by State Key Laboratory for Microbial Technology (Shandong University, China). It is also a nonionic biosurfactant, and one possible structure of sophorolipid from *Wickerhamiella domercqiae *analyzed by Chen et al. is given in Figure 1(b) [18].

All other chemical reagents, purchased from Kanto Chemical Co., Inc. (Japan) were of analytical grade. Water (>18.2 MΩ), which was treated using an ultrapure water system (Advantec aquarius: RFU 424TA), was employed throughout the work.

### 2.2. Samples

The original sludge sample was collected in May 2010 from an industrial water treatment plant. The sample was air-dried and removed coarse sand and stone, then ground and sieved through 120 mesh (0.125 mm).

The polluted sludge sample was prepared by adding the solution containing three kinds of metallic salts (NiCl_2_·6H_2_O (1000 ppm), Pb(NO_3_)_2_ (1360 ppm), and Cr(NO_3_)_3_·9H_2_O (1000 ppm)) to the air-dried original sludge sample. The polluted sample was shaken for 3 days on a shaker at room temperature (25.0 ± 0.2°C) and subsequently centrifuged at 3000 rpm for 30 min using a centrifugal separator (Kubota Co. 5200). The supernatant was discarded, and the polluted sludge was air-dried and sieved through 120 mesh (0.125 mm).

Basic characteristics of the sludge samples, such as pH, EC, moisture content, and cation exchange capacity (CEC) were measured, based on the method for soil testing recommended by The Japanese Geotechnical Society [19]. For measuring organic matter content, 10 g air-dried sludge samples were heated for 2 h at 105°C and then burned at 550°C in a furnace for 6 h. Organic matter content was estimated from the weight differences of the sludge before and after burning divided by the sludge weight before burning. Permeability is an important physical parameter to determine the feasibility of the soil flushing process. Therefore, the permeability of the sludge was also determined using the Unfirmed Water Head Test [20]. The specific surface area of each sample was measured using Micrometritics TriStar3000. The BET method and Langmuir method, as well as methods used in our previous work, were applied to determine the surface area [21, 22].

 For measuring total metal concentration, the sludge was digested with HNO_3_-HF by using the microwave digestion method as well as the case of digesting soil [23]. After this, the analysis of metallic elements was performed using ICP-AES or ICP-MS.

### 2.3. Distribution of Metallic Elements in Sludge

 All heavy metals including REEs, Th, and U in sludge samples were partitioned into six fractions with sequential extraction procedures mainly based on Sadamoto et al. [24] and Tessier et al. [25]. In this paper, these six fractions, (1) water soluble, (2) exchangeable, (3) bound to carbonates, (4) bound to organic matter, (5) bound to Fe-Mn oxides, and (6) residual were, denoted as F1, F2, F3, F4, F5, and F6, respectively. The sequential extraction procedure is outlined in Table 1. For the initial step in this sequential extraction procedure, 7 g of dried sludge sample in 100 cm^3^ polypropylene centrifuge tube was used. Following extraction in each step, the mixture of sludge sample and each extraction reagent was centrifuged (3000 rpm × 30 min) using a centrifugal separator (Kubota Co. 5200). This procedure is the same used in our previous work on soil [26]. The concentrations of metallic elements in each fraction were determined with ICP-AES or ICP-MS.

### 2.4. Batch Test and Column Test

The effect of the concentration and pH value of biosurfactant solution on the removal of heavy metals in polluted sludge was investigated in batch experiments at room temperature (25°C). Each 1.0 g of contaminated sludge was weighed into a centrifuge tube, and 25 cm^3^ of biosurfactant solution, varying in initial concentrations from 1 to 50 g·dm^−3^, was added to each tube. The tubes were then shaken in a reciprocating shaker for 24 hours to attain equilibrium. The suspension was centrifuged (3000 rpm × 30 min) using a centrifugal separator (Kubota Co. 5200). The supernatant solutions were separated and dissolved with 1 mol·dm^−3^ HNO_3_ after digestion for analysis. Subsequently, the effect of pH value (varied from 2.5 to 6.5) was investigated with the same procedure as above.

Column tests were also conducted to remove heavy metals from polluted sludge at the optimum concentration and pH of the biosurfactant solution. Permeability is a useful parameter for the flushing techniques like column experiments. Silica was mixed with polluted sludge (mass ratio of 4 : 1) to be compacted in column to improve the low permeability of sludge. 30 g of each mixture was packed in a glass column (internal diameter of 1.5 cm and length of 30 cm) and two in total. Saponin solution and ultrapure water were prepared for mobilization and leaching of heavy metals from sludge at less than a rate of 0.2 cm^3^·min^−1^ using multichannel peristaltic pump. Each leachate was collected per one flush volume and then was digested and dissolved in 1 mol·dm^−3^ HNO_3_ for analysis.

### 2.5. Recovery of Heavy Metals from Leachates

The precipitation method was applied by using 3 mol dm^−3^ hydroxide sodium (NaOH) [27]. The leachate from polluted sludge from washing with saponin was used as a sample after determining the concentration of heavy metals. The pH value of the leachate was gradually increased because heavy metals were precipitated as hydroxide. The solution was allowed to stand for 24 hours before being centrifuged with a refrigerated centrifuge, after which, the concentration of heavy metal was measured with ICP spectrometry.

## 3. Result and Discussion

### 3.1. Characteristics of Sludge

 Some physical-chemical characteristics of sludge have been determined (Table 2). As shown in this table, the pH value of the polluted sludge is lower than that of the original sludge, whereas EC is remarkably large. These results may be attributable to the fact that the polluted sludge was prepared by adding a solution containing three kinds of metallic elements (Pb, Ni, and Cr). Furthermore, CEC of the sludge became small after the introduction of heavy metals. It is known that soil flushing proves effective only for permeable soil (*K* > 1.0 × 10^−3^ cm·s^−1^) or, to a lesser extent, slightly permeable soil (1.0 × 10^−5^ cm·s^−1^ < *K* < 1.0 × 10^−3^ cm·s^−1^) [28]. The permeability of the sludge studied in this work (*K≈* 1.7 × 10^−5^ cm·s^−1^) is much lower than the value of permeable soil, so quartz sand was added into the sludge to improve its permeability in column washing experiments. From the above mentioned, it is perhaps obvious that the pH, EC, and CEC values were changed by the introduction of metals.

### 3.2. Concentrations and Distribution of Heavy Metals in Sludge

The concentrations of heavy metals (Zn, Pb, Cd, Ni, Cr, and Cu) found in the sludge are listed in Table 3. The relative standard deviation (RSD) of the triplicated analyses of each sample was less than 5%. From Table 3, the concentration of Zn was the highest, and the total concentration of Pb, Ni, Cr, and Cu did not exceed the limits in “The Criterion about the Waste Including Metals” [29], but Cd is relatively high in the original sludge. For polluted sludge, the concentrations of uncontaminated elements (Zn, Cd, and Cu) were almost unchanged; however, the concentrations of contaminated element increased remarkably. These results indicate that most of lead, chromium, and nickel in solution have been introduced successfully into the sludge. The absorption rates of Pb, Ni, and Cr in this study were 86.5%, 68.4%, and 93.1%, respectively, which may be mainly attributed to competitive sorption onto the sludge. These results concur with those reported by Juwarkar et al. [30].

**Figure 2:**
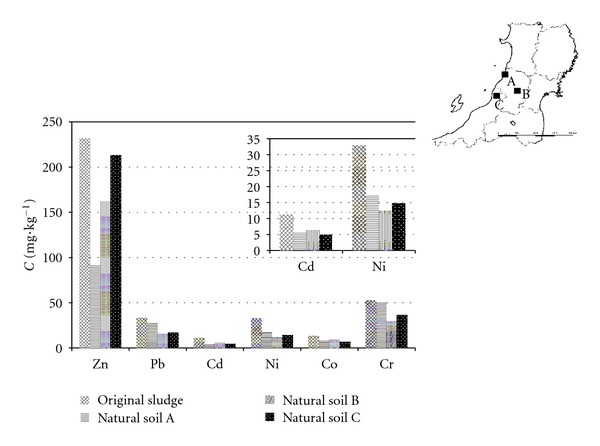
The concentrations of some heavy metals (Zn, Pb, Cd, Ni, Co, and Cr) in original sludge and natural soil ((A) Sakata City and (B) Murayama City in Yamagata Prefecture in Japan and (C) Sekikawa Village in Niigata Prefecture in Japan).

For reference, the concentrations of heavy metals in natural soil are also shown in Figure 2 along with those of the original sludge. It was found that the concentrations of heavy metals in sludge are higher than those in natural soil [26] (natural soil used in this work is no plow soil from Ueno, Sekikawa village in Niigata Prefecture, Toyasato, Sakata town and Tateoka, Murayama town in Yamagata Prefecture, resp.). One possible reason for high concentrations in sludge is that the sludge was mainly precipitated from wastewater (containing many kinds of heavy metals), which is discharged from the industries such as paper manufacturing, petrochemical engineering, glass production, textiles, and transportation. In particular, the concentrations of Cd and Ni in sludge are markedly higher (up to double) than those in natural soil. This suggests that heavy metals in sludge may tend to accumulate in agriculture soil if the sludge is used repeatedly.

**Figure 3:**
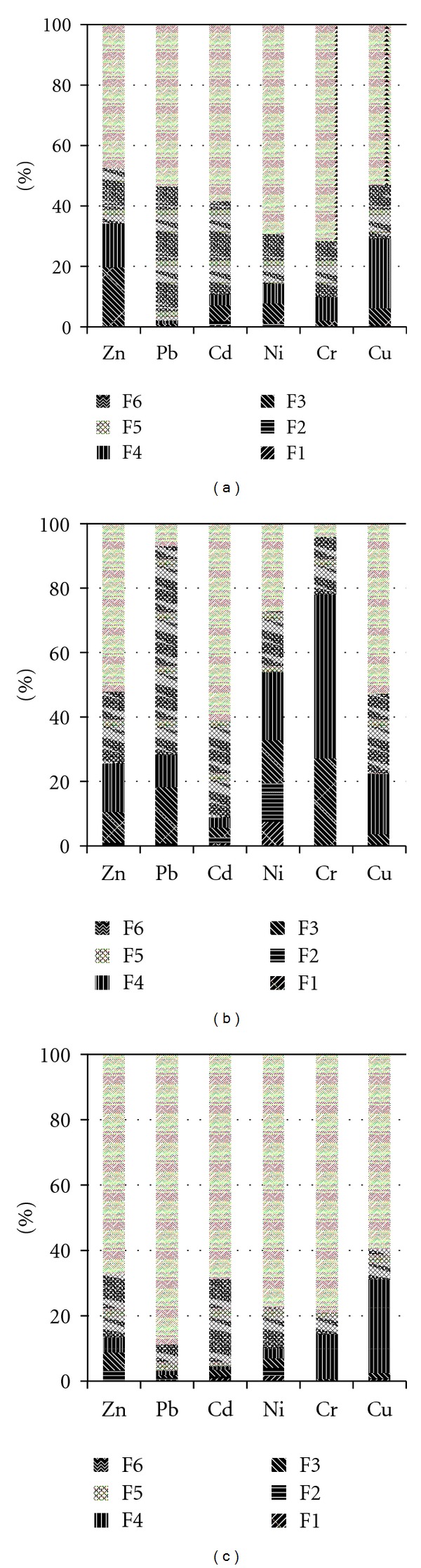
The relative distribution of some heavy metals (Zn, Pb, Cd, Ni, Co, and Cr). (a) Original sludge. (b) Polluted sludge. (c) Natural soil C.

The relative distribution of heavy metals is shown in Figure 3. The results in Figure 3(a) suggested that, in addition to the residual fraction, Pb, Cd, Ni, and Cr mainly exist as Fe-Mn oxides fraction, Zn exists as carbonate fraction, and Cu exists as organic fraction in original sludge. These results are in accordance with the distribution characteristics of heavy metals in soil [26, 31, 32].

Comparing Figure 3(a) with Figure 3(b), the following results can be obtained. (1) The concentration of Ni in F2 (bound to exchangeable fraction) sharply increased, which suggests that Ni may be very harmful to the environment at the beginning period of pollution. (2) Heavy metals (Pb, Ni, and Cr) were in relatively unstable fractions (from F1 to F5) at the early stage of pollution and generally moved to the stable residual fraction (F6) with time and become difficult to remove from soil. Considering this, the remediation of polluted soil by heavy metals should be carried out as soon as possible. (3) The dominant fraction (except residual fraction, F6) is different among elements; that is, the order of the relative distributions is “oxide fraction” > “carbonate fraction” > “organic fraction” for Pb, and “organic fraction” > “carbonate fraction” > “oxide fraction” for Cr. Ni does not show any dominant chemical fraction although the general tendency is “organic fraction” > “oxide fraction” > “carbonate fraction” > “exchangeable fraction.” It indicates that Pb is easily adsorbed to Fe-Mn oxides and that Cr is drawn to organic matter in sludge. (4) The dominant fraction of uncontaminated elements (i.e., Zn, Cd and Cu) hardly changed, although the relative distribution of F3 (bound to carbonate fraction) decreased in case of Zn.

Comparing Figures 3(a) and 3(c), the proportion of residual fraction (F6) in natural soil is relatively higher than that in original sludge. In contrast, the proportions of oxide fraction (F5) and carbonate fraction (F3) in original sludge are higher than those in natural soil. From these results, it is found that heavy metals in natural soil usually exist in a more stable state than those in sludge. It indicates that it may be hard for heavy metals in natural soil to permeate into groundwater or to be absorbed by crops. On the other hand, the high proportion of oxide fraction in sludge may be due to relative large contents of Fe and Mn in sludge (Table 4).

In brief, the concentrations of heavy metals in sludge are larger than those in natural soils. The relative distribution of residual fraction in natural soil is higher than that in original sludge, while the ratio of oxide fraction in natural soils is lower than that in original sludge.

### 3.3. Concentrations and Distribution of REEs, Th, and U in Sludge

**Figure 4:**
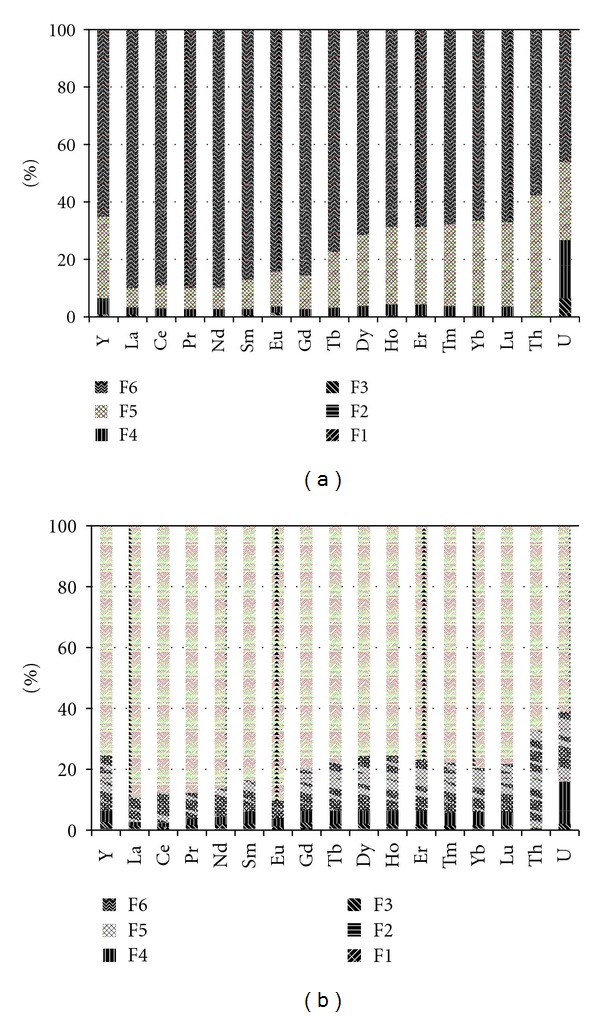
The relative distribution of REEs, Th, and U. (a) Original sludge. (b) Natural soil C.

 REEs, Th, and U were also extracted from the sludge along with heavy metals and determined with ICP-MS. The concentrations are shown in Table 5, and the relative standard deviation (RSD) of the triplicated analyses of each sample was less than 10%. The relative distribution of REEs, Th, and U is shown in Figure 4 (distribution characteristics of REEs, Th, and U in natural soil A and B are similar to those in natural soil C, so the data for soil A and B are not shown in this figure.). Judging from Table 5, the concentrations of REEs in the sludge are similar to those in natural soil C, while the concentrations of Th and U are smaller than those in natural soil C. On the other hand, the concentrations of metallic elements (except for HREE, i.e., heavy rare earth elements) in sludge are higher than those in natural soil A and B.

Figures 4(a) and 4(b) show that the distribution characteristics of REEs, Th, and U are generally similar to those of heavy metals. It is noted that, except for U which is higher, the proportion of carbonate fraction (F3) in sludge is lower than that in natural soil. These results show that REEs, Th, and U in natural soil exist in more stable states than those in sludge. In addition, high carbonate fraction of U in sludge is noticeable because the available content in crops is generally considered to be reflected, to some extent, by metal content in carbonate fraction [33].

As mentioned above, the concentrations of REEs, Th, and U in sludge are variable when compared with those in natural soils. Furthermore, heavy metallic elements including REEs, Th, and U in sludge exist as more unstable fraction than those in natural soil.

### 3.4. Removal of Heavy Metals in Sludge

 The removal of heavy metals (Pb, Ni, and Cr) in polluted sludge was investigated with elution technology by using biosurfactant (nonionic biosurfactant sophorolipid and saponin). To quantify the factors influencing the removal efficiency of biosurfactant, the effects of the concentration and pH value of the biosurfactant solution in batch experiments and the washing volume of the biosurfactant solution in column experiments were tested in this work.

#### 3.4.1. Batch Experiments

**Figure 5:**
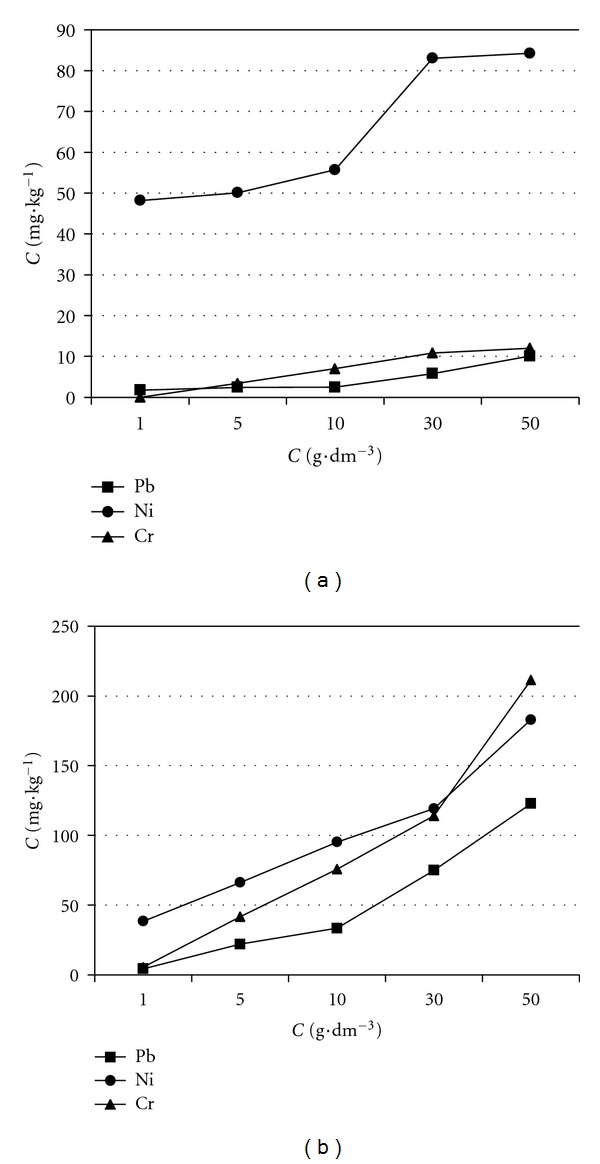
Effect of concentrations on removal of heavy metals by batch experiments with biosurfactant as washing agent: (a) sophorolipid and (b) saponin.

**Figure 6:**
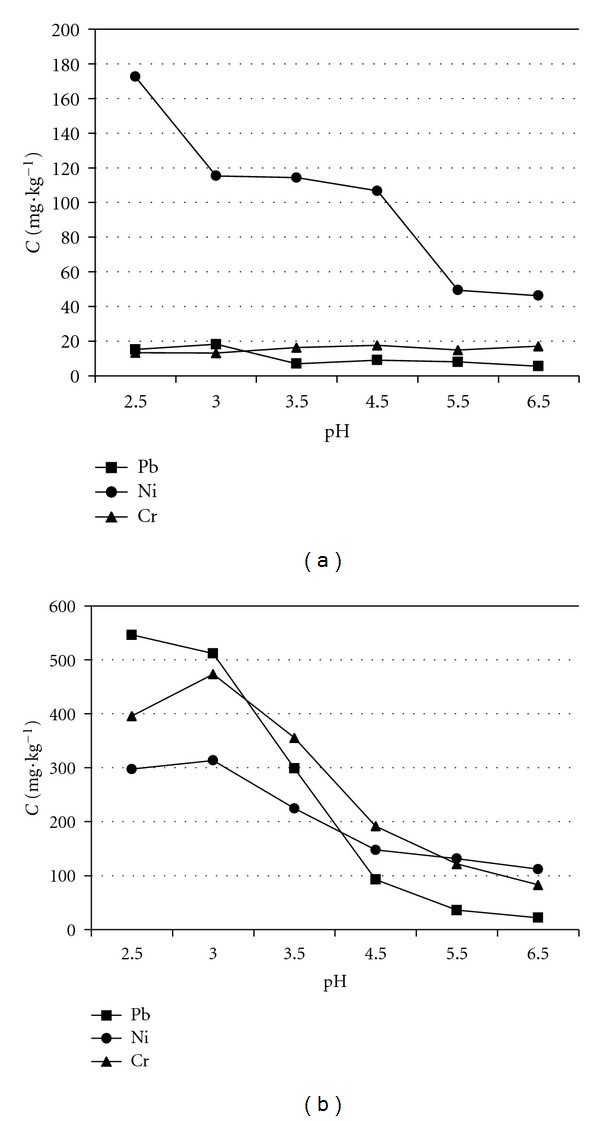
Effect of pH value on removal of heavy metals by batch experiments with biosurfactant as washing agent: (a) sophorolipid and (b) saponin.

The effects of the concentrations of the biosurfactants solution on the removal efficiency of heavy metals are shown in Figure 5, and the effects of pH value of the biosurfactants solution on the removal efficiency of heavy metals are shown in Figure 6. For both biosurfactants, the concentration ranged from 1 to 50 g·dm^−3^, and pH ranged from 2.5 to 6.5. The removal efficiency of heavy metals by both biosurfactants generally ascended with increasing concentration and decreasing pH value; however it was clear that saponin is more efficient than sophorolipid. Although both biosurfactants are nonionic, the saponin used in this work contained the carboxyl group in sapogenin moiety [17]. For this reason, saponin reacts more easily with metallic elements and to make metallic elements depart from the sludge surface into the soil solution. Because of this only the results using saponin are discussed in the following.

The removal efficiency is the greatest when the concentration of saponin solution (i.e., 50 g·dm^−3^) is the highest (Figure 5(b)). However, the sludge is apt to produce colloidal precipitation due to the adsorption of biosurfactant molecules when the concentration is 50 g·dm^−3^. Because of this an optimum saponin solution concentration of 30 g·dm^−3^ was selected for the following column experiments.

Figure 6(b) shows that the removal efficiency is dependent on pH. When the pH value of saponin solution is higher than its pKa (4.6), the removal efficiency is low. It may be considered that sodium ions, which increased by adding NaOH to adjust pH of saponin solution, compete with heavy metals for saponin. In contrast, when the pH value was lower than its pKa, the removal efficiency abruptly increased. However, when the pH value was less than 3.0, the removal efficiency of Ni and Cr was reduced. This may be due to the amount of saponin adsorbed onto sludge, which increased with decreasing pH because electrostatic attraction between saponin and sludge surface increases at low pH [27]. For this reason a pH of 3.0 was applied in the following column experiments.

#### 3.4.2. Column Experiments

**Figure 7:**
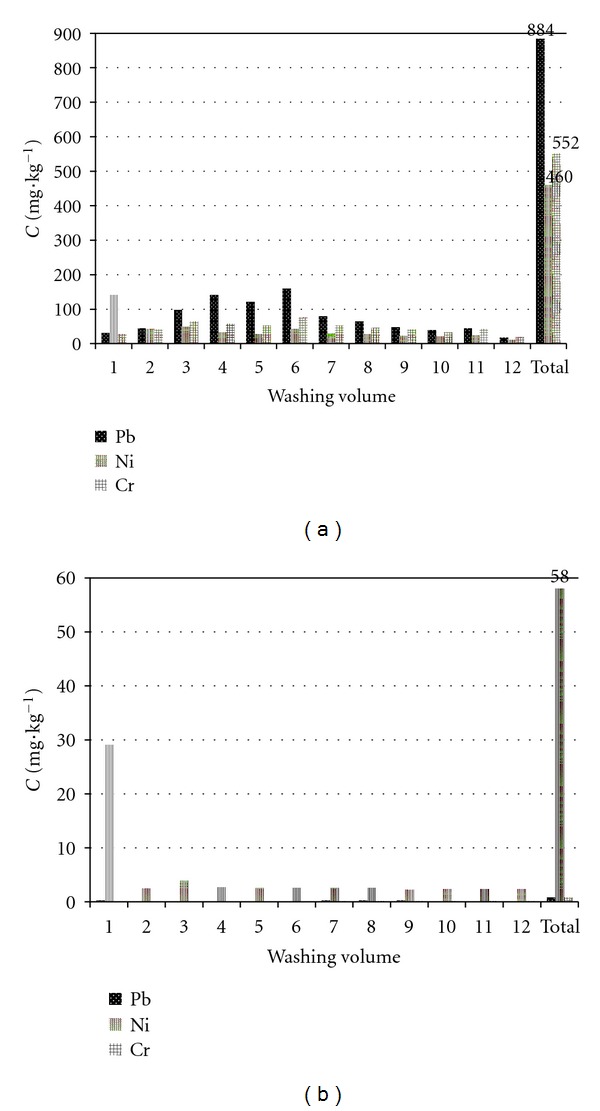
Effect of washing volume on the removal of heavy metals by column experiments: (a) saponin and (b) ultrapure water (1 w.v. (washing volume) = 6.2 dm^3^).

 The concentrations of heavy metals removed from the polluted sludge with washing volume through column are illustrated in Figure 7. In addition to biosurfactant solution, ultrapure water was used as eluent for the control. As seen in Figure 7(a), the removal of each metal showed a peak with the increasing of washing volume. In the case of Pb, Ni, and Cr, 884, 460, and 552 ppm, respectively, were removed overall from total loaded concentration.  Figure 7(b) shows that, except some removal of Ni (58 ppm), hardly any metals were removed with ultrapure water. From Figures 7(a) and 7(b), it is found that saponin has high potential for the removal of heavy metals from polluted sludge compared to ultrapure water.

After 12 washing volumes (one washing volume is about 6.2 cm^3^), the total removal efficiency reached 73.2%, 64.2%, and 56.1% for Pb, Ni, and Cr, respectively. The results indicate that saponin facilitates mobilization of metals selectively and that the leaching behavior of biosurfactant is dependent on the characteristics of the metals. This may be due to the specificity of biosurfactant for each metal and the coexistence of metals in the sludge.

#### 3.4.3. Confirmation of Fraction Removed by Saponin Solution

**Figure 8:**
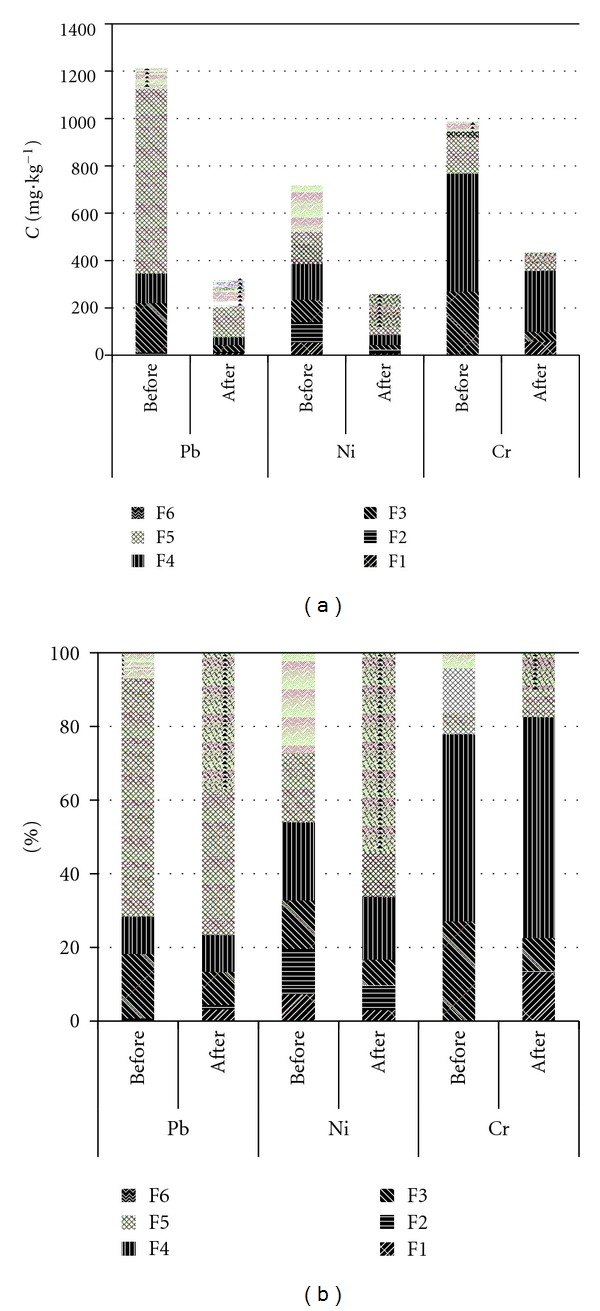
The concentrations and the relative distribution of heavy metals in polluted sludge before and after the column experiments: (a) concentration and (b) relative distribution.

To confirm the fractions of heavy metals removed by column flushing with saponin solution, sequential extraction was conducted after the column washing. The concentrations of heavy metals (Pb, Ni, and Cr) in polluted sludge before and after the column washing are shown in Figure 8(a), and the relative distribution of each heavy metal is shown in Figure 8(b). Figure 8(a) shows that a remarkable decrease of total concentration was found for each heavy metal and that the concentration in each fraction was also changed regardless of the kind of metal. The concentration of F1 for Pb and Cr slightly increased due to the residual saponin in the sludge, which can further react with heavy metals in the extraction process. The concentrations of three elements in F3, F4, and F5 all decreased. Of the three fractions, however, F4 showed the smallest decrease. It may be that heavy metals in F3 and F5 could be more easily released than those in F4 under acidic conditions (pH 3). For the same reason, it is suggested that the removal efficiency of Cr (the proportion of this element in F4 was over 50% of total concentration) was the lowest among the three kinds of metals. From Figure 8(b), it is found that the proportion of the relative stable fraction of heavy metals became higher after column washing and that the relative distribution characteristics of heavy metals was closer to that in natural soil.

From the above mentioned, saponin is more efficient than sophorolipid for the removal of heavy metals from sludge in this work. Saponin has selectivity for the mobilization of heavy metals and mainly reacts with the F3 and F5 fractions of heavy metals.

### 3.5. Recovery of Heavy Metals from Sludge

**Figure 9:**
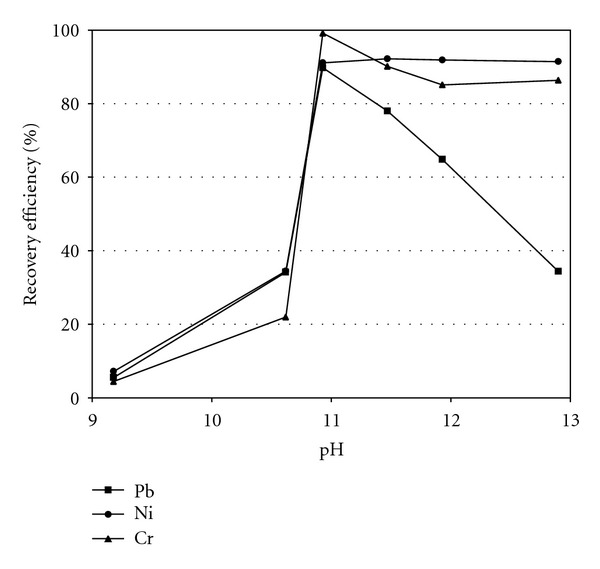
The recovery efficiency of heavy metals from the sludge leachate.

In order to recover heavy metals from the sludge leachates, the precipitation method by adding NaOH was firstly considered.  Figure 9 shows the recovery efficiency of heavy metals from the sludge leachate at pH 9.2–12.9 using the precipitation method. At pH 10.9, the recovery efficiency of each heavy metal almost reached the maximum possible and was 89.7%, 91.1%, and 99.1% for Pb, Ni, and Cr, respectively. Due to the amphoteric nature of lead and chromium, their hydroxide compounds (i.e., precipitate) are redissolved, and this decreased the recovery efficiency (in addition to other issues such as the waste of alkaline solution) at excessively high pH (i.e., >11.5). Therefore, the optimal pH for recovery was considered to be about pH 10.9. That is to say, it is an effective method to use an alkaline solution for obtaining high recovery efficiency of heavy metals such as Pb, Ni, and Cr.

Although most of Pb, Ni, and Cr in the polluted sludge was removed with saponin by washing in the column, the residual concentrations are still higher than those in agricultural soil. Even still, this work has quantitatively shown that, to some extent, saponin could be an efficient sorbent for the removal of heavy metals from sludge. However, further investigations to survey the method for improving the removal efficiency of heavy metals, to elucidate the mechanism of the removal of heavy metals by surfactant, and to survey the selection of the optimum surfactant and the optimum conditions for the removal of heavy metals are needed in future research.

## 4. Conclusion

The behavior, distribution, and characteristics of heavy metals including REEs, Th, and U in sludge from an industry water treatment plant were investigated and compared with those in natural soil. Furthermore, the removal/recovery process of heavy metals (Pb, Cr, and Ni) from the polluted sludge was studied with biosurfactant elution by batch and column experiments. Consequently, the following conclusions have been obtained.

1. The concentrations of heavy metals in sludge are greater than those in natural soils, and the concentrations of REEs, Th, and U in sludge are variable when compared with those in natural soils. The relative distribution of the residual fraction in natural soil is higher than that in original sludge. On the other hand, the relative distribution of oxide fraction in original sludge is higher than that in natural soils. That is, heavy metallic elements in sludge have generally greater concentrations and exist as more unstable fraction than those in natural soil.
2. Nonionic saponin is more efficient than sophorolipid for the removal of heavy metals from sludge. Saponin has selectivity for the mobilization of heavy metals and mainly reacts with F3 and F5 fractions of heavy metals. In other words, nonionic biosurfactants including the carboxyl group have high potential for the removal of heavy metals in sludge.
3. The recovery efficiency of heavy metals (Pb, Ni, and Cr) reached about 90–100% by the precipitation method with alkaline solution.

## Figures and Tables

**Table 1:** Sequential extraction procedure for fractional determination of metallic elements.

Step	Fraction	Extraction reagents	Ratio of sludge : reagent	Extraction condition
1	F1	Extrapure water	7 : 70	Shake 6 h, 30°C
2	F2	0.05 mol·dm^−3^ Ca(NO_3_)_2_	7 : 70	Shake 24 h, 30°C
3	F3	2.5% CH_3_COOH	7 : 70	Shake 24 h, 30°C
4	F4	6% H_2_O_2_	7 : 120	Water bath, 95°C (Evaporate)
		2.5% CH_3_COOH	7 : 70	Shake 24 h, 30°C
5	F5	0.1 mol·dm^−3^ (COOH)_2_ + 0.175 mol·dm^−3^ (COONH_4_)_2_ + Ascorbic acid	7 : 210	Water bath, 6 h, 95°C Occasional shaking
6	F6	HNO_3_ + HF		Microwave digestion

**Table 2:** Physical-chemical characteristics of original sludge and polluted sludge.

Parameters	Original sludge	Polluted sludge
pH(H_2_O)	5.78	4.47
pH(KCl)	5.08	4.42
EC, *μ*S·cm^−1^	360	1926
Moisture content, %	6.10	7.13
Permeability, cm·s^−1^	1.75 × 10^−5^	1.72 × 10^−5^
Organic matter content, %	14.0	14.0
Cation exchange capacity (CEC), cmol·kg^−1^	33.6	30.5
BET-specific surface area (SSA), m^2^·g^−1^ Langmuir-specific surface area (SSA), m^2^·g^−1^	36.0 56.2	29.4 45.8

**Table 3:** The concentrations of heavy metals in sludge (mg*·*kg^−1^).

	Zn	Pb	Cd	Ni	Cr	Cu
Original sludge	232	33.5	11.2	32.7	53.0	50.7
Polluted sludge	233	1.21 × 10^3^	11.5	716	984	51.2

**Table 4:** The concentrations of Fe and Mn in original sludge and natural soil (mg*·*kg^−1^).

	Original sludge	Natural soil A	Natural soil B	Natural soil C
Fe	4.73 × 10^4^	2.69 × 10^4^	3.71 × 10^4^	3.20 × 10^4^
Mn	769	694	936	618

**Table 5:** The concentrations of REEs, Th, and U in original sludge and natural soil (mg*·*kg^−1^).

	La	Ce	Pr	Nd	Sm	Eu	Gd	Tb	Dy	Ho	Er	Tm	Yb	Lu	Th	U
Original sludge	22.3	48.6	5.54	28.6	5.13	1.28	6.40	0.764	3.75	0.728	2.18	0.297	1.90	0.283	7.90	2.33
Natural soil A	12.9	26.5	2.89	11.5	2.44	0.86	2.51	0.386	2.31	0.463	1.42	0.195	1.33	0.192	4.46	0.911
Natural soil B	14.5	31.8	3.67	15.0	3.46	1.21	3.94	0.644	3.46	0.708	2.19	0.308	2.14	0.321	4.09	1.31
Natural soil C	21.5	47.1	5.16	19.4	4.06	0.718	3.68	0.566	3.02	0.578	1.74	0.241	1.71	0.248	23.4	4.18
